# Inhibition of IL1R1 or CASP4 attenuates spinal cord injury through ameliorating NLRP3 inflammasome-induced pyroptosis

**DOI:** 10.3389/fimmu.2022.963582

**Published:** 2022-08-05

**Authors:** Chenfeng Wang, Hongdao Ma, Bangke Zhang, Tong Hua, Haibin Wang, Liang Wang, Lin Han, Qisheng Li, Weiqing Wu, Yulin Sun, Haisong Yang, Xuhua Lu

**Affiliations:** ^1^ Department of Orthopaedics, Shanghai Changzheng Hospital, Shanghai, China; ^2^ Department of Anesthesiology, Shanghai Changzheng Hospital, Shanghai, China

**Keywords:** spinal cord injury, NLRP3 inflammasome, pyroptosis, IL1R1, CASP4, GSDMD, gasdermin D

## Abstract

Spinal cord injury (SCI) is a devastating trauma characterized by serious neuroinflammation and permanent neurological dysfunction. However, the molecular mechanism of SCI remains unclear, and few effective medical therapies are available at present. In this study, multiple bioinformatics methods were used to screen out novel targets for SCI, and the mechanism of these candidates during the progression of neuroinflammation as well as the therapeutic effects were both verified in a rat model of traumatic SCI. As a result, CASP4, IGSF6 and IL1R1 were identified as the potential diagnostic and therapeutic targets for SCI by computational analysis, which were enriched in NF-κB and IL6-JAK-STATA3 signaling pathways. In the injured spinal cord, these three signatures were up-regulated and closely correlated with NLRP3 inflammasome formation and gasdermin D (GSDMD) -induced pyroptosis. Intrathecal injection of inhibitors of IL1R1 or CASP4 improved the functional recovery of SCI rats and decreased the expression of these targets and inflammasome component proteins, such as NLRP3 and GSDMD. This treatment also inhibited the pp65 activation into the nucleus and apoptosis progression. In conclusion, our findings of the three targets shed new light on the pathogenesis of SCI, and the use of immunosuppressive agents targeting these proteins exerted anti-inflammatory effects against spinal cord inflammation by inhibiting NF-kB and NLRP3 inflammasome activation, thus blocking GSDMD -induced pyroptosis and immune activation.

## Introduction

Spinal cord injury (SCI) is a common nervous system disease which may cause permanent neurological dysfunction (1), with approximately 180,000 new cases diagnosed per year in the world (2). Despite advances in surgical techniques and efforts dedicated to the treatment of SCI, there is currently no effective regimen to treat this devastating neurological disease (3, 4). Given the seriousness of this disease, a comprehensive understanding about the pathomechanism of SCI is primarily important in mining the potential targets and searching for effective treatment strategies.

Continuous progress and development of bioinformatics have made it possible to explore underlying mechanisms of multiple diseases at genetic and molecular levels, and provide guidelines for better treatment. Bioinformatical analysis has demonstrated that hub genes are expressed differentially across the acute, subacute and chronic phases of SCI, which offers some meaningful insights into different pathomechanisms and targeted therapies between acute and chronic SCI (5). Besides, weighted gene co-expression network analysis (WGCNA), as an important bioinformatics method, can assist to explore the underlying mechanism of SCI-induced immune suppression (6). Furthermore, leveraging the benefits of bioinformatics is an innovative way to mine cell markers and immune responses following SCI (7).

Interestingly, an increasing number of studies have proven that immune cell infiltration plays a key role in the advancement of SCI. γδ T cells, a subgroup of T cells, could be recruited into the SCI site for exacerbating inflammation and impeding neurological self-impair *via* CCL2/CCR2 signaling (8). Neutrophils are reported to play an essential role in the progression of SCI, and recruitment of macrophages at the lesion site is regarded as a potential approach for treating SCI (9, 10).

The motivation of this study was to explore key biomarkers of SCI as well as obtaining the therapeutic targets for SCI therapy with the help of bioinformatical analysis and experiments. First, multiple bioinformatics methods were used to screen out novel targets for SCI. Second, the mechanism of these candidates during the progression of neuroinflammation was verified in a rat model of traumatic SCI. Finally, the therapeutic effects of immunosuppressive agents targeting biomarkers for SCI treatment were clarified.

## Materials and methods

### Data collection

Three transcription datasets GSE5296 (11), GSE47681 (12) and GSE45006 (13) were downloaded from the GEO database (https://www.ncbi.nlm.nih.gov/geo/). The dataset GSE5296 and GSE47681 based on GPL1261 [Mouse430_2] Affymetrix Mouse Genome 430 2.0 Array. The former dataset included 21 murine normal spinal cord and 75 SCI samples, and the latter one included nine normal and 25 SCI samples. To evaluate the efficiency of analysis, the GSE45006 dataset was utilized as the validation set using GPL1355 [Rat230_2] Affymetrix Rat Genome 230 2.0 Array as the platform, which contained four normal samples and 20 SCI samples.

### Obtainment of DEGs

To remove the inter-batch difference, GSE5296 and GSE47681 were integrated through the “affy” package (14) and the “sva” package (15), showing in a two-dimensional PCA cluster plot (16). The obtainment of DEGs depended on the “limma” package (17), exhibiting on a volcano figure with the usage of the “ggplot2” package (18). DEGs with p < 0.05 and |log_2_FC|>1 were viewed as statistically significant.

### Functional enrichment analysis and PPI network

To obtain biological functions and signaling pathways for disease, the Metascape database (www.metascape.org ) was used for functional annotation. Gene Ontology (GO) and Kyoto Encyclopedia of Genes and Genomes (KEGG) pathway enrichment analyses were performed for specific genes. Min overlap ≥3 and p ≤0.01 were considered statistically significant.

All the genes were input into the STRING (http://string−db.org) online database for investigating the interaction between proteins and constructing their network. Among the high confidence (score 0.900), a ‘tsv’ file was imported into the Cytoscape software to visualize the network.

### Acquisition and validation of diagnostic markers

LASSO logistic regression (19) and Boruta algorithm (20) were applied to conduct feature selection to screen key markers for SCI. LASSO logistic regression, a machine learning strategy, finds λ to decide the variables when the classification error is littlest. LASSO regression allows the coefficients of the features to be compressed by a penalty function to obtain the optimal constrained model; this approach avoids the overfitting and covariance found in classical analysis methods and also enhances the generalization ability of the model. The Boruta algorithm is a random forest classifier package that finds all the relevant feature variables. They are often utilized to search characteristic variables and assemble an excellent classification model. After quality control, the expression matrices of the GSE5296, GSE47681, and GSE45006 datasets were merged into an independent dataset for the next analysis. The LASSO logistic regression was utilized through the “glmnet” package (21). Additionally, the Boruta algorithm is a feature-selection algorithm that randomly disrupts the order of each real feature, evaluates the importance of each feature, and iteratively removes features of low relevance to find the best variable. In this study, “Boruta” package (20) was used for feature selection, and a total of 500 trees were constructed to a deeper level of verification for the diagnostic efficiency of these biomarkers. The overlapping genes obtained by these two algorithms are considered as signature genes and they are validated in GSE45006. Three receiver operating characteristic (ROC) curves were generated to estimate the accuracy of the screened biomarkers. The area under the curve (AUC) represents the performance of the model.

### Correlation analysis of genes and immune cell infiltration

The CIBERSORT algorithm is widely used to assess immune cell types in various diseases. In this study, data related to spinal cord injury were analyzed using CIBERSORT to infer the relative proportions of 22 immune infiltrating cells and to plot the associated heat map using the “corrplot” and “vioplot” packages. And Spearman correlation analysis was conducted for candidate markers and immune cells that participated in the progress of infiltration, which were visualized by using “ggplot2” package.

To evaluate the influence of genes on immune cell infiltration (22), ssGSEA (23) was used to quantify the infiltration level of immune cells in each sample.

### Gene set variation analysis (GSVA)

GSVA (24) is a non-parametric and unsupervised method for assessing gene set enrichment (GSE) and identifying important signaling pathways involved in SCI. The corresponding genomes were downloaded from the Molecular Signature Database (http://gsea-msigdb.org) and the GSVA R package was used to find differential expression pathways and biological processes between high and low expression groups of candidate genes.

### Antibodies and reagents

The antibodies and reagents used in this study included anti-IL1R1 (Abclonal, A5727), anti-GAPDH (Proteintech, HRP-60004), anti-CASP4 (Affinity, AF5130), anti-cleaved CASP4 (Affinity, AF5373), anti-IGSF6 (Abclonal, A15128), anti-phospho-NF-κB pp65 (Servicebio, GB13025), anti-Bcl2 (Abclonal, A0208), anti-CASP3 (Abclonal, A2156), anti-IL1B (Abclonal, A16288), anti-NLRP3 (Affinity, DF7438), anti-ASC (Abcam, ab180799), anti-GSDMD (Cell signaling technology, 39754), anti-cleaved GSDMD (Cell signaling technology, 10137), anti-NF-κB p65 (Cell signaling technology, 3033), Goat anti-rabbit IgG-HRP (Proteintech, HRP60004), Goat anti-rabbit pp65 (Servicebio, GB21303), TUNEL (Servicebio, GB1501), DAPI (Servicebio, G1012), Anakinra (MCE, AMG-719), and Belnacasan (Selllock, also known VX-765, S2228).

### Establishment of the rat SCI model

SD male rats aged 60 days and weighing 180-200g were used to build the SCI model. Briefly, after successful anesthesia with 1% pentobarbital, laminectomy was performed to expose the spinal cord at T10 level, and then a spinal cord impactor (F69852, RWD, CA, USA) was utilized to make an injury by dropping a 5g rod onto the spinal cord from a height of 6.5 cm. After the operation, the animals were carefully nursed, fed and pressed to promote urination three times a day until the bladder reflex was recovered. Rats in the Sham operation group underwent laminectomy only at the same level.

### Basso Beattie Bresnahan (BBB) scale and footprint analysis

BBB scale was used to assess the hindlimb motor function of the rats on day 1, 3, 7, 14, 21, and 28 post-injury in an open field. After the rats had adapted to the environment, two well-trained experimenters observed and scored the locomotor function in 5 minutes, and the mean BBB score of three measurements was used for analysis.

Footprint analysis was performed by dipping the rat hind paws in dye. All rats were allowed to walk across a narrow box measuring 1 m long and 7 cm wide, and the footprints were scanned.

### Swimming test

The swimming test is also a scoring system to evaluate functional recovery. All rats were trained to swim from one end to the other end of a water-filled glass tank, and their swimming strokes were scored by the Louisville Swim Scale (LSS) in terms of forelimb dependency, hind limb movement and alternation, trunk instability, and body angle. Each rat was tested twice to calculate the mean score.

### Drug administration and animal grouping

Anakinra is an inhibitor of IL1R1, while VX-1765 is an inhibitor of CASP4. As no inhibitor for IGSF6 was available, it was excluded in the subsequent experiments. Forty-eight rats were randomly divided into four groups: a Sham group, a SCI+normal saline (NS) group, a SCI + Anakinra (SA) group, and a SCI + VX765 (SV) group. Anakinra in SA group was injected intrathecally at a dose of 25μg/5μl on the day of modeling as it was done in SV group. In Sham and NS group, 5μl NS was injected intrathecally on the day of modeling.

### Tissue collection

According to the protein expression time curves of the three biomarkers (IL1R1, CASP4 and IGSF6), follow-up experiments were carried out to further verify the accuracy of the signatures. After experimental verification, the injured spinal cord tissues processed in different groups were collected on day 7 after injury. The rats were anesthetized by 1% pentobarbital and perfused with 0.9% saline (containing 50 U/mL heparin) through the endocardium and then perfused with phosphate buffer (containing 4% PFA). A 10-mm spinal cord segment was cut at the injured site, fixed in 4% PFA for 48 h at room temperature, and paraffin-embedded.

### Haematoxylin-eosin (HE) staining

The fixed, dehydrated and paraffin-embedded tissues were sliced into 4-μm sections. After deparaffinization and hydration with gradient ethanol, they were stained with hematoxylin for 5 min, differentiated with 1% hydrochloric acid and ethanol, and blued with 5% ammonia. The sections were stained with 0.5% eosin for 1 min, dehydrated and sealed with neutral glue. Pathological changes of the injured spinal cord were observed under an optical microscope.

### Immunohistochemistry

The paraffin-embedded tissues were sliced and incubated in 0.3% H2O2 for 30 min, and then in 0.1% Triton X-100 for 20 min. Next, the sections were incubated with primary antibodies, including anti-IL1R1 antibody (1:100), anti-IGSF6 antibody (1:100), and anti-CASP4 antibody (1:100) overnight at 4°C and secondary antibody for 60 min at 37°C. Finally, the sections were stained with DAB for color development and counterstained with hematoxylin.

### Immunofluorescence staining

Cryosections of the spinal cord tissue were rinsed with 0.01 M PBS, and then blocked with 5% normal goat serum, 0.1% bovine serum albumin, and 0.2% Triton-X 100 in 0.01 M PBS before applying the primary antibody of rat anti-pp65 (1:100). Target signals were visualized by using rabbit HRP with fluorescence-conjugated secondary antibodies. The TUNEL kit was used to stain at 37°C for 2 h, and confocal images were obtained using a scanning instrument (Pannoramic DESK P-MIDI P250, 3D HISTECH, Hungary).

### Western blot analysis

The proteins of the spinal cord (epicenter ± 5mm) were extracted by protein extraction buffer (Beyotime, P00103J) supplemented with 1% protease inhibitor (Beyotime, P1005) in a tissue grinding machine (Servicebio, KZ-III-FP). Cell lysates were centrifuged at 12000g for 15min at 4°C to collect supernatants. The protein concentration was determined by the BCA kit (Beyotime, P0012) and followed by denaturing at 95°C for 10 min in 1×SDS loading buffer. Subsequently, samples with an equal amount of protein were loaded and applied to 10% SDS-PAGE and then transferred onto nitrocellulose membranes (Millipore). The membranes were sealed and then incubated with specific primary and secondary antibodies. Proteins were visualized using enhanced chemiluminescence substrate (Tanon) and then quantified using a Tanon Chemiluminescent Imaging System.

### Real-time quantitative PCR (RT-qPCR) analysis

Through machine grinding, total RNA of cracking organizations was extracted using the Trizol reagent (Vazyme) and then converted to cDNA using a reverse transcription kit HiScript II Q RT SuperMix for qPCR (R122-01, Vazyme, China). Next, RT-qPCR was performed using AceQ qPCR SYBR Green Master Mix (Q111-02, Vazyme, China) in a 7500 real-time PCR system (Applied Biosystems, Inc., USA) according to the manufacturer’s instruction. The primer sequences used for RT-qPCR are listed in **Table 1**. The mRNA levels of the target genes were normalized to the Glyceraldehyde-3-phosphate dehydrogenase (GAPDH) expression. Quantification of the RT-qPCR results was performed by the 2ΔΔCt method.

**Table 1:** Primer sequences for RT-qPCR.

Gene	Forward	Reverse
GAPDH	GACATGCCGCCTGGAGAAAC	AGCCCAGGATGCCCTTTAGT
CASP4	CTTACGGCTGAGGGCATGGAATC	CAAGTGGTGTGGTGTTGTAGAGTAGAG
IGSF6	ACAGCAATCCAAACAACAGCAAAGAG	GACAGTTACTTCCGCTCTGCCTTC
IL1R1	TTGTCTCATTGTGCCTCTGCTGTC	GCTGATGAATCCTGGAGTCCTTGTC

### Statistical analysis

The statistical analysis was conducted in R v3.6. All statistical tests were bilateral, and p < 0.05 was considered statistically significant. Data between two groups were compared by an unpaired t-test, and comparisons between multiple groups were performed with one-way analysis of variance (ANOVA) whereas pairwise comparison within groups was conducted by *post hoc* test.

## Results

### Normalization and DEGs obtainment

**Figure 1:**
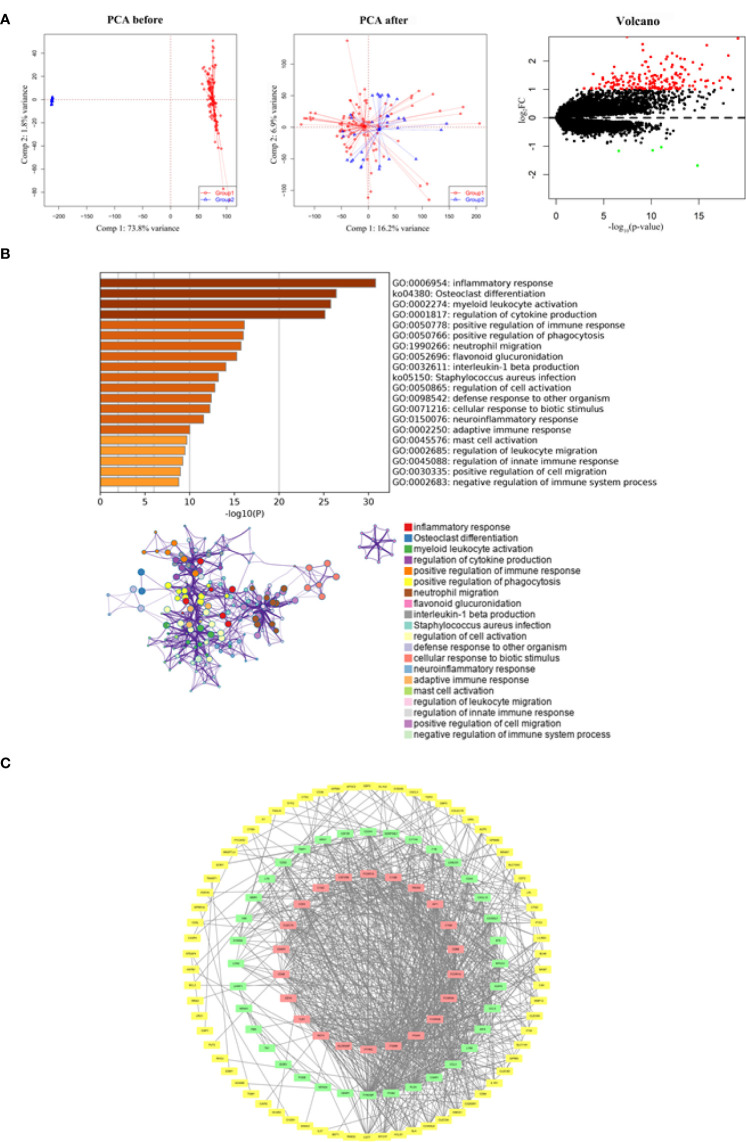
Functional enrichment and PPI network analysis of identified DEGs. **(A)** Two-dimensional PCA cluster plot of GSE5296 and GSE47681 datasets before and after normalization: group1 represented the SCI group and. group2 represented the normal control group. Red dots represent up-regulated genes, black dots represent no significant difference, and green dots represent down-regulated genes. **(B)** GO enrichment analysis demonstrated the main biological process and signaling pathways. **(C)** The PPI network construction. The red squares construct the most significant cluster, the green squares represent other clusters, and the yellow squares represent the genes not involved in cluster construction.

After merging the GSE5296 and GSE47681 datasets, the newly generated gene expression matrix needed processing, which was presented in a two-dimensional PCA cluster layout before and after processing, suggesting that the aid of samples used was reliable. A total of 182 DEGs were identified, including 178 up-regulated genes and four down-regulated genes (**Figure 1A**).

### Functional enrichment analysis and PPI network analysis

By understanding the signaling pathways, biological processes and interactions involved in DEGs, it is an important step to uncover the pathological mechanisms of SCI. Functional enrichment analysis showed that DEGs were associated with inflammatory response, osteoclast differentiation, myeloid leukocyte activation, regulation of cytokine production, and positive regulation of immune response (**Figure 1B**). Of the 128 nodes and 934 edges, DEGs that did not participate in the construction of PPI network were either hidden or deleted (**Figure 1C**).

### Acquisition and validation of diagnostic markers

**Figure 2:**
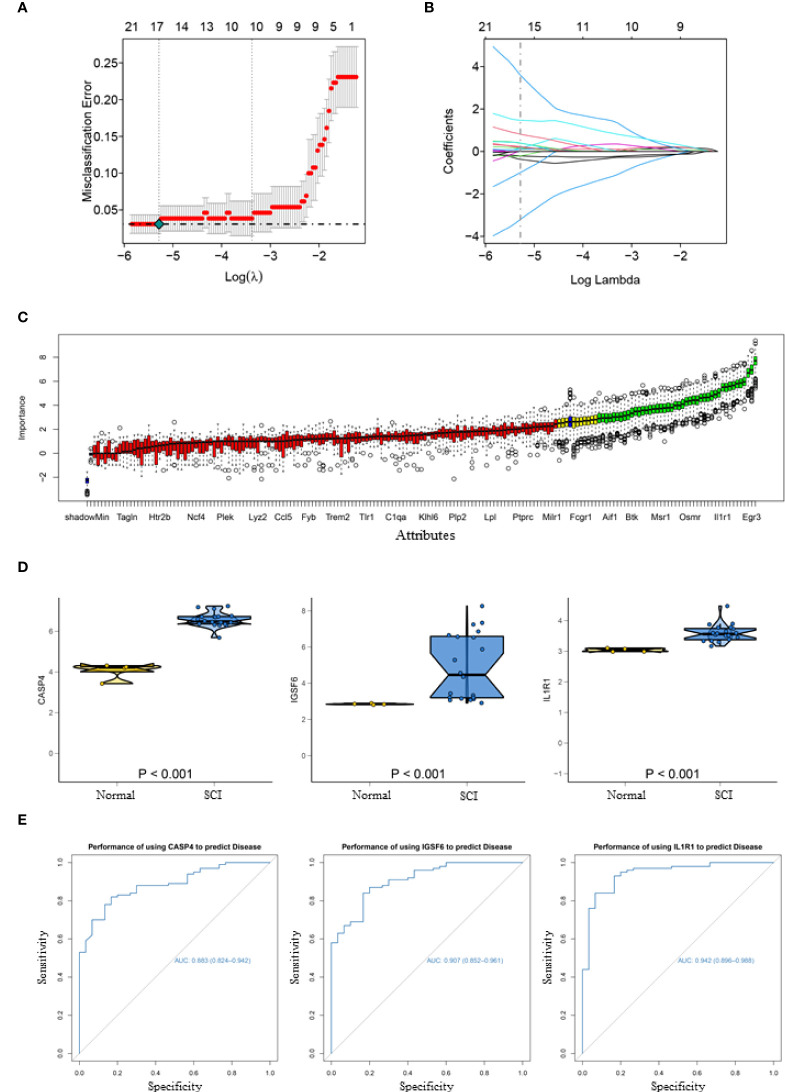
Acquisition and validation of candidate diagnostic markers. **(A)** LASSO regression showed the binomial deviation of different quantitative variables. The lower horizontal coordinate indicates the value of the lasso regression model parameter λ, the upper horizontal coordinate indicates the number of coefficients in the corresponding model, and the vertical coordinate indicates the error of the model. There are two dashed lines, the line with the lowest error on the left and the line with fewer features on the right. **(B)** Coefficient of LASSO regression model. As the λ value changes, the later the coefficient is compressed to zero the more important the variable is. **(C)** Boruta algorithm to screen diagnostic biomarkers. **(D)** Box violin maps of CASP4, IGSF6 and IL1R1 indicate that the differential expression in SCI group was significantly different from that in the normal group. **(E)** The ROC curves of the diagnostic efficacy of CASP4 (AUC=0.883), IGSF6 (AUC=0.907) and IL1R1 (AUC=0.942), the area under which was used to evaluate the effectiveness.

To obtain more representative biomarkers, we select two feature selection algorithms and validate the results. 15 DEGs were identified as biomarkers for SCI by LASSO logistic regression (**Figures 2A, B**) and 43 by the Boruta algorithm (**Figure 2C**). By overlapping the feature genes, we finally obtained eight feature genes (SLA、IL1R1、NPAS4、CASP4、IGSF6、EGR3、NR4A1 and NAIP5). As described before, GSE45006 was used to further verify the intersecting genes, finding that CASP4 (caspase 4), IGSF6 (immunoglobulin superfamily member 6), and IL1R1 (interleukin 1 receptor type 1) varied significantly in SCI. Three genes were differentially expressed between the normal and SCI samples, which are respectively presented in a box violin map (**Figure 2D**). In addition, the ROC curve of diagnostic efficacy demonstrated that CASP4 (AUC=0.883), IGSF6 (AUC=0.907), and IL1R1 (AUC=0.942) had a high diagnostic value (**Figure 2E**). IL1R1 and CASP4 are involved in non-classical pathways of pyroptosis process (25), and as for IGSF6, it plays a significant role in the nerve system (26).

### Correlation analysis between biomarkers and infiltrating immune cells

**Figure 3:**
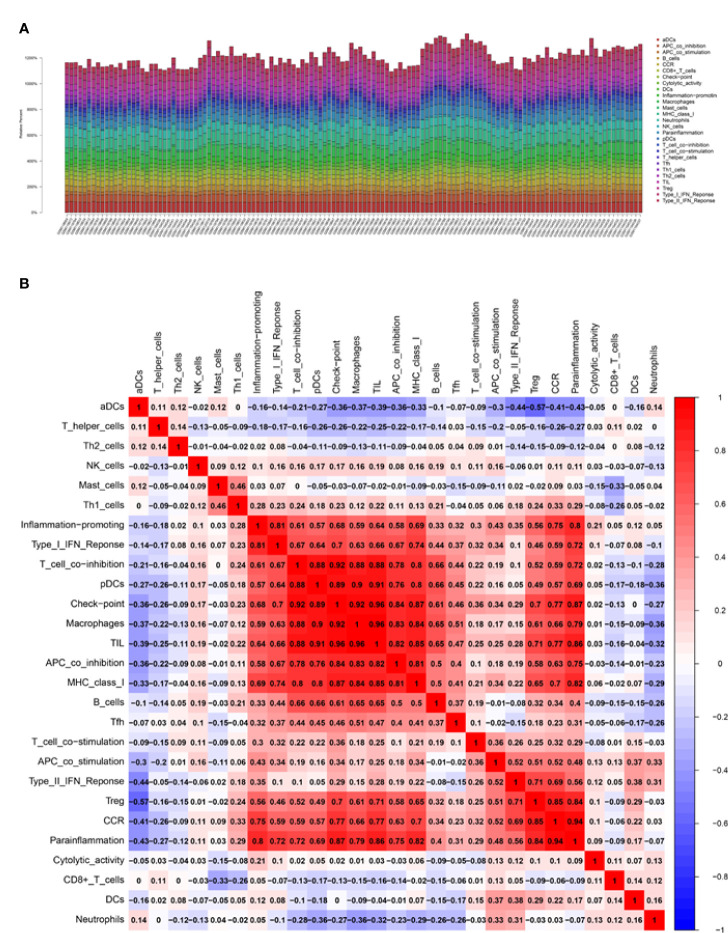
Correlation analysis of the biomarkers and infiltrating immune cells. **(A, B)** Relative immune cells and responses in 130 samples from GSE5296 and GSE47681 datasets.

**Figure 4:**
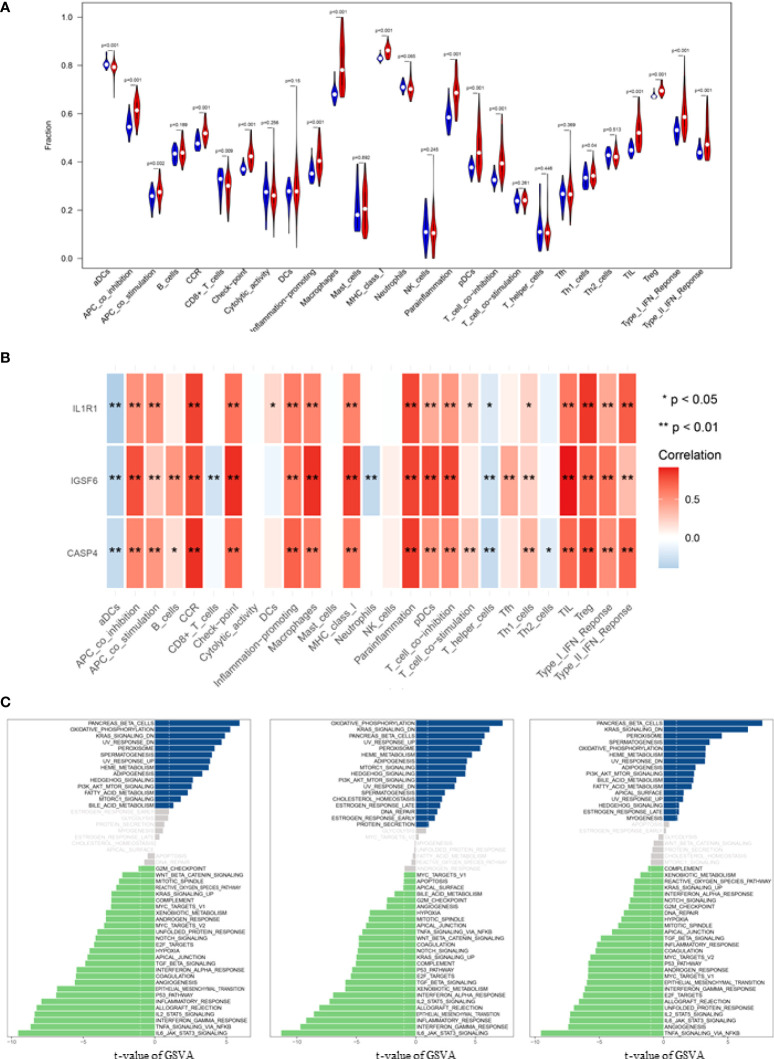
Correlation analysis of infiltrating immune cells and Gene set variation analysis (GSVA). **(A)** Differences in immune infiltration between SCI and normal control groups. Blue represents the normal control group, and red represents SCI group. p-value <0.05 was considered statistically significant. **(B)** * p = < 0.05, ** = p < 0.01, and non-* = statistically insignificant. The color of the squares represents the closeness degree of the relationship between the genes and immune cells, the darker the color, the higher the p-value, and the lighter the color, the lower the p-value. **(C)** The GSVA results demonstrated the pathway differences of these key gene expressions in SCI.

It is known that immune cells contribute to the inflammatory responses in SCI progression. Therefore, we further explored the immune infiltration that is closely associated with SCI. With the help of the CIBERSORT, the difference in immune infiltration was summarized between 100 SCI and 30 normal spinal cord tissues in subpopulations of immune cells (**Figure 3A**). Compared with the control, the injured spinal cord contained a higher proportion of antigen-presenting cells (APC), macrophages, check-point, Treg, and tumor-infiltrating lymphocytes (TIL) (**Figures 3B**, **4A**).

Correlation analysis demonstrated that CASP4 was positively associated with chemokine receptor (CCR) and para-inflammation, and negatively associated with activated dendritic cells (aDCs); IGSF6 was positively associated with TIL and macrophage, and negatively associated with aDCs and neutrophils; IL1R1 was positively associated with Treg and Parainflammation, and negatively associated with aDCs (**Figure 4B**).

### GSVA analysis

The biological effects of the three biomarkers were evaluated using the GSVA algorithm, and the results demonstrated that there were significant differences in 33 common pathways, including activated pathways such as the IL6-JAK-Akt signaling pathway and TNFα signaling *via* NFκB (**Figure 4C**).

### IL1R1, CASP4, and IGSF6 are upregulated in the injured spinal cord

**Figure 5:**
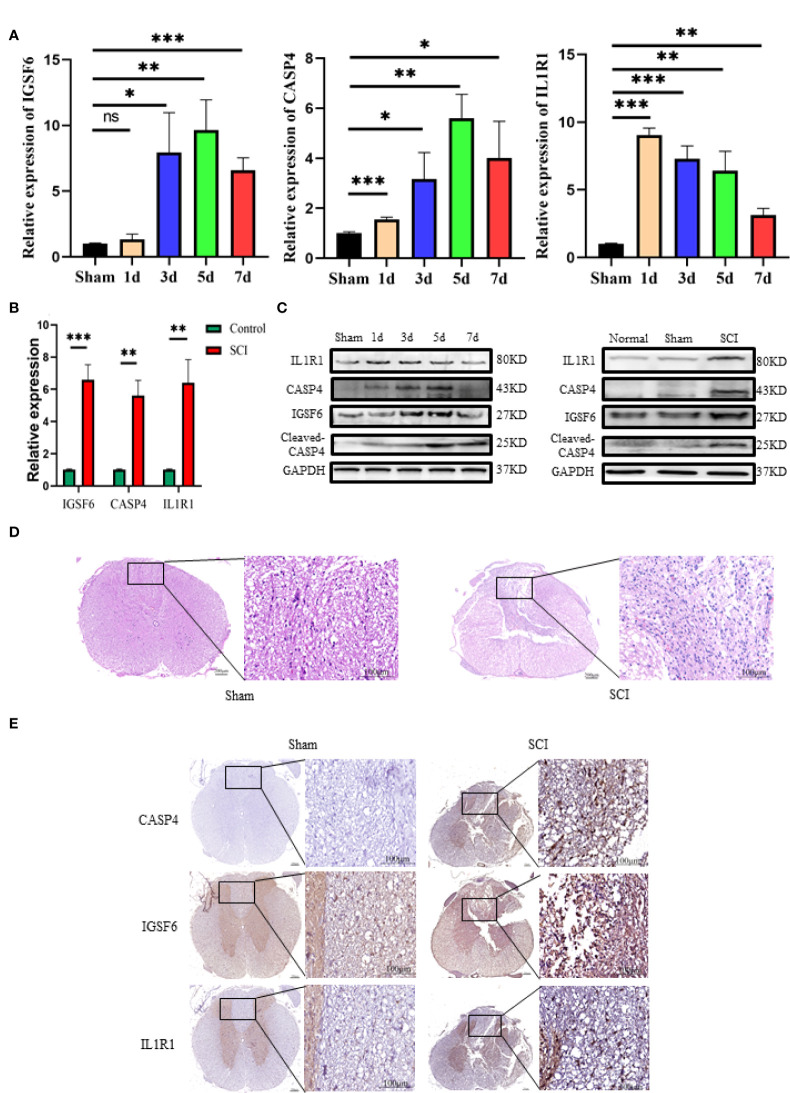
Western blot, RT-qPCR and immunohistochemistry of the three biomarkers (IL1R1, CASP4 and IGSF6). **(A)** The time curves of the three biomarkers at mRNA level on day 1, 3, 5 and 7 after SCI or sham surgery (n = 3 rats per group at each time point, values are the mean ± SD, *p < 0.05, **p < 0.01, ***p < 0.001, t-test). **(B)** RT-qPCR validation of the biomarkers expressing in peak: the three signatures were increased significantly in SCI group (n = 3 rats per group, values are the mean ± SD, *p < 0.05, **p < 0.01, ***p < 0.001, t-test). **(C)** Western blot validation of the biomarkers as represented by their time curves at protein level (n = 3 rats per group, values are the mean ± SD, *p < 0.05, t-test). **(D)** Histomorphologically, the injured spinal cord tissue exhibited organizational abnormalities as compared with the normal control (overall scale bars = 200μm, regional-scale bars = 100μm). **(E)** Immunohistochemical validation of the biomarkers: the markers were found to be expressed in both gray matter and white matter (Overall scale bars = 200μm, regional-scale bars = 100μm, the arrows point to the positioning of the marker).

To explore the role of the biomarkers in the pathophysiology of SCI, RT-qPCR and Western blot were used to determine their expression patterns in the lesion site at day 1, 3, 5 and 7 after injury. The results showed that the three biomarkers were upregulated in SCI (**Figures 5B, C**). The protein expression of IL1R1 was the highest at day 1 after injury, and both CASP4 and IGSF6 peaked at day 5 after injury. Besides, the mRNA expression of IL1R1 was significantly upregulated at day 1 after injury; IGSF6 and CASP4 reached the peak level at day 5 after injury, which was coincident with the protein expression (**Figure 5A**). The HE staining (**Figure 5D**) showed no significant pathological change in Sham group, while significant abnormalities in the gray and white matter boundary in SCI rats, including focal bleeding, necrosis and cavity formation. Immunohistochemistry showed that the expression of the three signatures increased markedly after SCI **(** **Figure 5E**).

### Locomotor functional recovery

**Figure 6:**
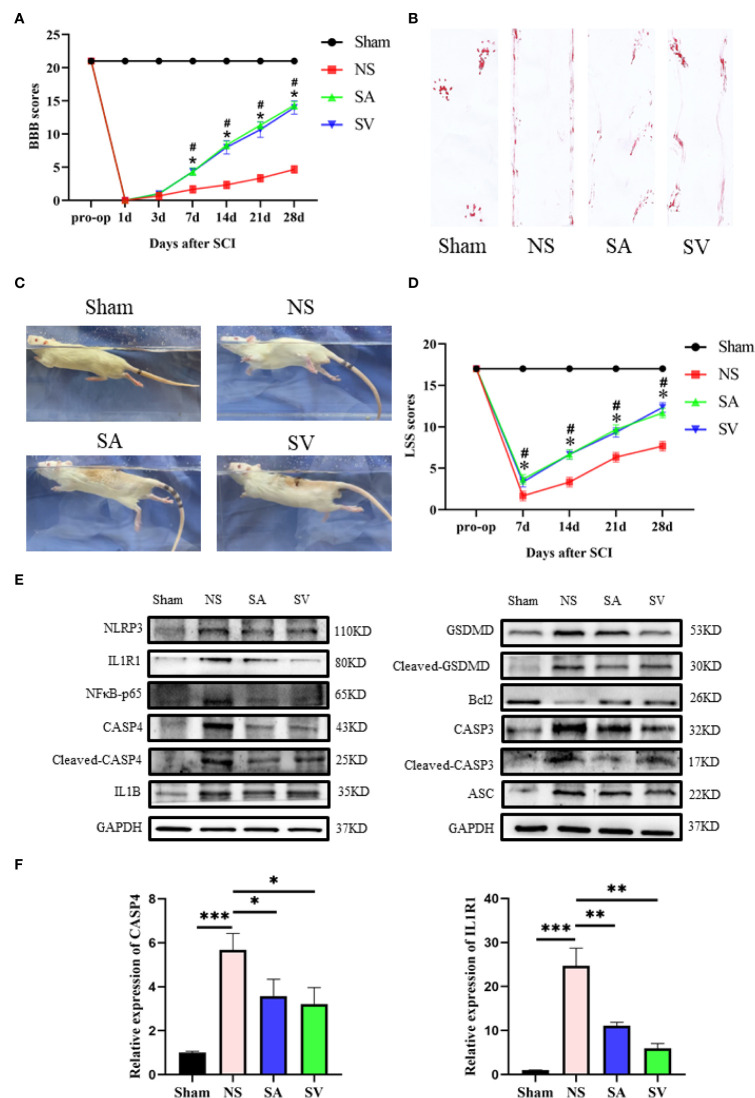
Change of the biomarkers and functional recovery in the SCI model after immunosuppressive therapy. **(A)** Statistical analysis of the BBB Scale in Sham, positive control, and treatment groups over 28 days (n = 3 rats per group at each time point, values are the mean ± SD, *p < 0.05, t-test). **(B)** Footprint analysis of different groups. **(C, D)** Statistical analysis of the Louisville Swim Scale in the four groups over 28 days (n = 3 rats per group at each time point, values are the mean ± SD, *p < 0.05, t-test). **(E)** Western blot showed that the expression level of the biomarkers in the treatment groups was between the levels of sham and control groups, and the levels of Bcl2 and CASP3, NFκB, GSDMD, IL1B, ASC, and CASP3 in SA and SV groups were also between those of Sham and control groups (n = 3 rats per group, *p < 0.05, **p < 0.01, ***p < 0.001, t-test). **(F)** RT-qPCR showed that the level of biomarkers was significantly decreased after treatment (n = 3 rats per group, *p < 0.05, **p < 0.01, ***p < 0.001, t-test).

To validate the potential therapeutic value of the biomarkers, immunosuppressive therapy was performed against the selected targets by selecting appropriate doses of the inhibitors to treat SCI rats for a week to observe the long-term effect. The BBB scale, footprint experiments, and LSS were used to evaluate neurological functional recovery. As indicated in **Figures 6A, D**, there was no significant difference in BBB and swimming test scores between the four groups before SCI. Yet, the result of locomotor function assessment in SA and SV groups was significantly superior to that in NS group from day 7 after SCI. In addition, the LSS showed that the SCI rats receiving inhibition therapy exhibited less forelimb dependence, faster hind limb alternation, and a smaller angle between the body and water surface from day 7 after injury (**Figures 6C, D**). Footprint analysis at day 28 showed that rats in SA and SV groups exhibited consistent coordination of the hindlimbs and reduced toe dragging (**Figure 6B**). On the contrary, no such coordination ability was observed in NS group rats; rather toe dragging was obvious. These findings indicate that the targetable inhibitors could promote the therapeutic effect on the functional improvement of SCI rats.

### IL1R1 and CASP4 inhibitors attenuate inflammation and promote repair of the injured spinal cord

**Figure 7:**
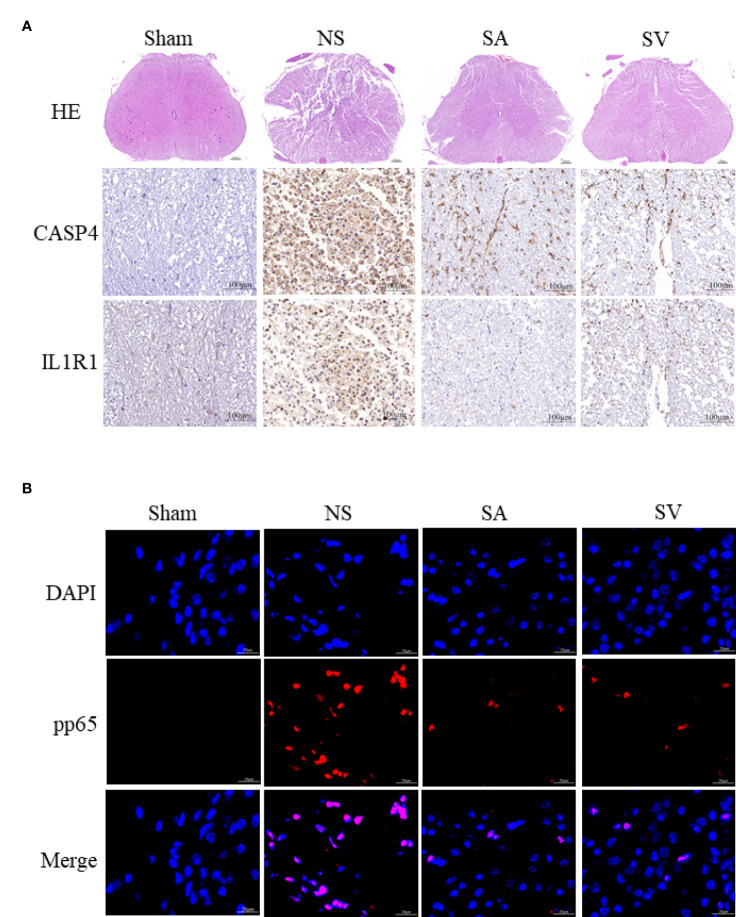
Histopathological changes. **(A)** HE staining demonstrated that the structure of the injured spinal cord tissue became clearer after treatment, with fewer cavities and necroses as compared with SCI group (Scale bars = 100μm, the arrows point to cavities and necroses), and the expression level of the biomarkers in the spinal cord was reduced (Scale bars = 20μm) (n = 3 rats per group, *p < 0.05, **p < 0.01, ***p < 0.001, t-test). **(B)** Representative images showing pp65 and DAPI co-staining of the spinal cord sections from the rats of the four groups (Scale bars = 20μm) (n = 3 rats per group, *p < 0.05, **p < 0.01, ***p < 0.001, t-test).

The use of IL1R1 and CASP4 inhibitors to reduce the degree of inflammation of secondary SCI and eliminate the cascade reaction may have a positive effect on SCI recovery. NLRP3, GSDMD, ASC, NFκB, IL1B and CASP4 are known to be related to pyroptosis, and Bcl2 and CASP3 are associated with apoptosis. Western blot analysis revealed that the levels of NLRP3, NFκB, GSDMD, IL1B, ASC and CASP3 in treatment groups were lower in SA and SV groups than NS group, while the Bcl2 level in SA and SV groups was significantly higher than that in NS group, which further proved that pyroptosis and apoptosis were involved in the cascade responses after SCI. With respect to the signatures, the expression of CASP4 and IL1R1 was in coincidence with other inflammatory and apoptosis indicators (**Figure 6E**). Meanwhile, the RT-qPCR of the three biomarkers illustrated that their mRNA expressions were significantly decreased in SCI rats after the inhibitor therapy (**Figure 6F**). Furthermore, the inhibitory effect on the expression of the biomarkers was detected by immunohistochemistry. It was found that the expression of two biomarkers was markedly decreased as represented by obvious attenuation of the staining area in SA and SV groups (**Figure 7A**).

**Figure 8:**
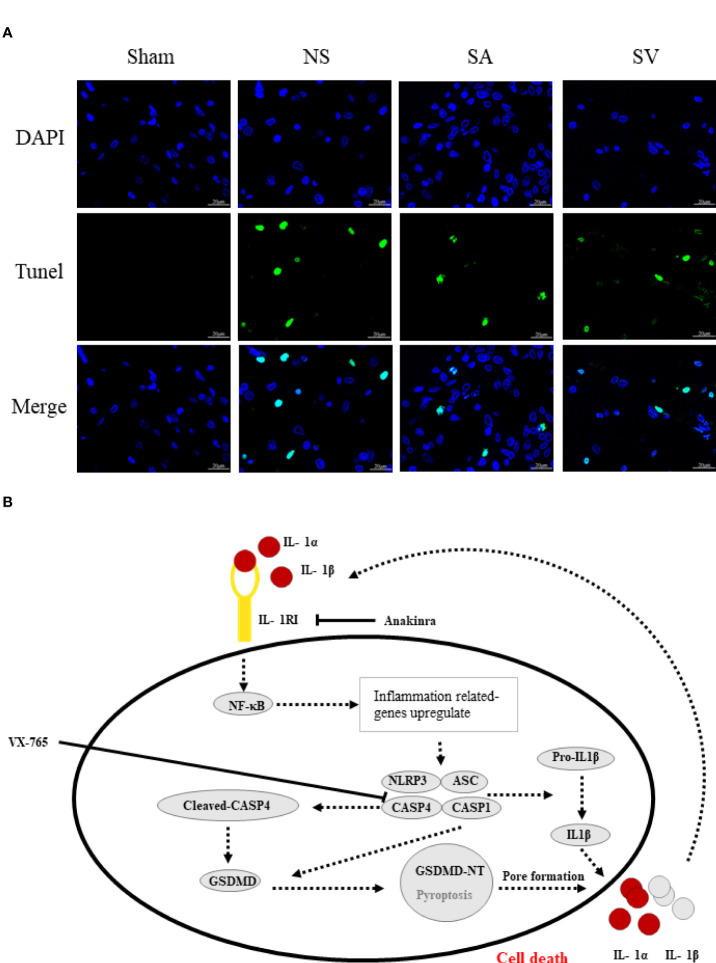
The therapeutic effect of the targetable drugs and exploration of the mechanism of spinal cord injury based on the biomarkers. **(A)** Verification of the therapeutic effect of Anakinra and VX765 for SCI by TUNEL staining (Scale bars = 20μm) (n = 3 rats per group, *p < 0.05, **p < 0.01, t-test). **(B)** Speculation for the pathomechanism of SCI.

As shown i**n** **Figure 7B**, the phosphorylation-NFκB (pp65) showed red dots, and the nucleus showed blue dots. Compared with Sham group, p65 was activated and then entered the nucleus, turning pink. However, P65 fluorescence expression in SA and SV groups was between Sham and NS groups, showing a small proportion of p65 activation (**Figure 7B**). Furthermore, immunofluorescence staining also showed that targetable treatment significantly decreased the number of TUNEL+ cells (**Figure 8A**). Collectively, our results suggested that Anakinra and VX765 utilization inhibited inflammation, decreased cell apoptosis and rehabilitated the physical function of the animals *in vivo*.

To observe the therapeutic effect of the drugs more directly, the spinal cord tissues were observed morphologically. It was found that the biopsy tissues in Sham group exhibited clear boundaries and an integrated morphology. In NS group, the structures of the spinal cord tissues were damaged histopathologically. Compared with NS group, such damage was dramatically reduced in SA and SV groups. The extent of focal hemorrhage and cavity in the gray matter of the treatment groups was mild and between that of Sham and NS groups (**Figure 7A**).

## Discussion

Spinal cord injury may cause nervous system dysfunction and remains a clinical challenge. Many studies have proposed that immune cells play a necessary role in the process of SCI. Therefore, exploration of related biomarkers, molecular mechanism and immune infiltration is indispensable for seeking advantageous treatment solutions and ultimately improving the quality of life of SCI patients. In this study, we employed bioinformatics analysis as an extensive approach to discover the biomarkers and CIBERSORT tools for the sake of finding out the immune cell infiltration patterns of the disease, and then verified the therapeutic effect of immunosuppressants targeting the markers on SCI. Finally, we identified a total of 182 DEGs that were enriched in inflammatory response, myeloid leukocyte activation, regulation of cytokine production, positive regulation of immune response, IL6-JAK-STAT3 signaling pathway, and TNFα signaling *via* NFκB. It was found that down-regulation of IL6-JAK-STAT3 and NFκB signaling pathways could inhibit inflammatory response and promote the recovery of neurological function after SCI (27, 28). Besides, we identified CASP4, IGSF6 and IL1R1 as the biomarkers of SCI by combining the LASSO logistic regression and Boruta algorithm and verified them by experiments. Delightedly, treating SCI with drug-targetable biomarkers could significantly improve the spinal cord morphology, inhibit GSDMD -induced pyroptosis and recover the behavioral ability. All these findings support our data analysis, confirming that the results obtained in this study are reliable and correct.

Some current studies have begun using machine learning and deep learning to select significant features and models. For example, as a learning machine, XGBoost can reliably predict neurological alterations, thereby enhancing personalized management and clinical prognosis of SCI patients (29). Besides, what counts is that effective machine learning can even precisely predict the prolonged ICU stay and the prolonged hospital stay to guide clinical treatment (30). LASSO logistic regression, a machine learning strategy, finds λ to decide the variables when the classification error is littlest. The Boruta algorithm is a random forest classifier package that finds all the relevant feature variables. They are often utilized to search characteristic variables and assemble an excellent classification model (31, 32). In this study, we identified CASP4, IGSF6 and IL1R1 as the biomarkers of SCI by combining the two algorithms.

The biomarkers were likely to relate to pyroptosis. CASP4, the ortholog of CASP11, as the receptor for cytoplasmic lipopolysaccharide, plays a role in the non-canonical inflammasome pathway and development of pyroptosis in SCI. CASP4 performs a similar function to CASP1, and its activation could induce GSDMD-dependent pyroptosis and processing of interleukin-18, further aggravating inflammatory damage (33). IGSF6 is a member of the immunoglobulin superfamily and is highly expressed in the immune tissue. It is considered as a candidate protein for inflammatory bowel disease susceptibility (34). Besides, IGSF6 is closely related to immune cell activity, which may participate in the progress of DC antigen presentation (35) and CD4^+^ T cell response (36). However, there is no study reporting the role of IGSF6 in SCI has not been reported and further experimental information is required. IL1R1, the ligand-binding receptor of IL1α and IL1β, can regulate IL1-related activities (37) and is highly expressed in SCI, which may contribute to posttraumatic inflammation response (38). However, IL1/IL1R1 is a double-edged sword, playing an essential role in aggravating or repairing the damage. Some SCI experiments suggested that astrocytes may initiate inflammation by promoting the entry of inflammatory monocytes and neutrophils in an IL1R1-dependent way (39). On the other hand, IL1/IL1R1 can exert beneficial effects, particularly in modest concentrations. For example, Parkinson’s disease progressed more rapidly in IL-1RI null mice as compared with their wild-type counterpart (40). Previous studies reported that CASP4, IGSF6 and IL1R1 may play significant roles in SCI and be considered as the biomarkers for SCI.

To gain deeper insights into the effect of immune infiltration in SCI, some studies used CIBERSORT to legitimately assess the relationship between immune cells and biomarkers. They found that CCR could early recruit monocytes to the lesion epicenter following SCI (41) and parainflammation response was closely related to the slow progress of SCI (42, 43). In addition, *in vitro* maturation of DCs was dramatically reduced in SCI patients (44). It was found that Tregs, an important factor in the immune system, could enhance repair of the injured nerve system by exerting an immunosuppressive effect through modulating multiple mechanisms (45). Cytokines are secreted by macrophages to regulate signaling ways to promote functional recovery after SCI (46). A study has shown that it is advantageous to decrease neutrophil activity to attenuate the pernicious impact of expanded neutrophil burst action (47). Hence, we conjecture that CASP4, IGSF6 and IL1R1 may participate in the occurrence and progression of SCI by increasing the positive-related cells or minimizing the negative-related cells. These suspicions need to be confirmed by more studies on the relationship between biomarkers and immune cells.

Mechanistically, pyroptosis involves two pathways: the canonical CASP1 inflammasome pathway, and the non-canonical CASP4/5/11 inflammasome pathway (48). The similarity between the two pathways is that both induce the maturation of GSDMD to complete pyroptosis, whereas the differences lie in the main components involved in the process of pyroptosis. In canonical pyroptosis centering around CASP1, After receiving stimulation, pro-CASP1 and ASC are recruited (often by NLRP3) to form inflammasomes, and then pro-CASP1 is cleaved to form caspase-1 for cleaving GSDMD (49). NLRP3 inflammasome, a sensor of damage-associated molecular patterns, plays a key role in SCI (50, 51). Our data showed that neuroinflammation caused by SCI increased the level of NLRP3 inflammasome, IL1β and GSDMD. Furthermore, NFκB subunit p65 will be activated by phosphorylation, promoting NLRP3 inflammasome activation. Based on the data currently available (25, 52), we made a speculation about the underlying mechanism (**Figure 8B**). Initiation of an inflammatory response increases the expression of various inflammatory cytokines such as IL1β and NLRP3. Post-SCI elevation of IL1R1 can activate NFκB signaling pathway by combining with IL1α or IL1β, which further upregulates inflammatory genes that encode IL1R1, pro- IL1α/IL1β and NLRP3. On the other hand, CASP4 that performs similar functions to CASP1 is activated to directly cleava GSDMD to initiate pyroptosis. Rupture of the cell membrane causes the release of inflammatory factors, which begins a new round of pyroptosis by binding to their receptors on the surrounding cell membrane. Interestingly, inhibition of IL1R1 could reduce the levels of pyroptosis-related proteins and apoptosis-related genes such as CASP3 and Bcl2. Inhibition of CASP4 could also decrease the expression of these genes, suggesting that drugs targeting these genes may be a potential strategy for the treatment of SCI by inhibiting the NLRP3 inflammasome formation and GSDMD -induced pyroptosis.

At present, the research on the application of immunosuppressants to SCI has made remarkable progress. Lee et al. (53) found that IL-20 antibodies improved motor function and reduced the formation of scar tissue around the injured spinal cord in rats with SCI, suggesting that IL-20 may be a potential therapeutic target for SCI. Giovanna Casili et al. (54) demonstrated that as an inhibitor of PARP-1/2, ABT888 exerts a protective effect after SCI by reducing autophagy-activating proteins and decreasing the apoptosis-autophagy mechanism. In contrast to these cases, the immunosuppressants we used were obtained by bioinformatic analysis of gene sequencing results, which is clearly directed; secondly, the progression of SCI can be detected by the altered expression of markers during the use of immunosuppressants, which in turn can better guide the application of the corresponding inhibitors; Besides, Anakinra has stable performance and significant therapeutic effect that has been applied to clinical studies of various diseases (55–57), and is expected to be a potential drug for the treatment of spinal cord injury. Yet, Anakinra and VX-765 have not yet been carried out to treat clinical patients with SCI, and further study is needed.

The reliability of the analysis results will be improved after introducing new calculation methods. Innovative scientific methods such as the Boruta algorithm and LASSO logistic regression were employed to prove biomarkers of SCI. As it is difficult to obtain large numbers of human spinal cord samples, we used mouse and rat samples instead, which may cause inaccuracy in genomic analysis. Since the focus of this paper is to explore the reliability of biomarkers and the effectiveness of marker-targeted immunosuppressants, we will perform cellular experiments to explore underlying mechanisms and functional phenotypes in the next step to further demonstrate our results.

In conclusion, CASP4, IGSF6 and IL1R1 were identified as key biomarkers of SCI. Inhibition of these biomarkers was regarded as an effective approach to achieving SCI repair by inhibiting NF-kB and NLRP3 inflammasome activation, thus blocking GSDMD -induced pyroptosis. Besides, TIL, dendritic cells, Treg, macrophages, parainflammation, and neutrophils may play essential roles in the progression of SCI. These findings may help mine the potential mechanism and explore treatment options for SCI.

## Data availability statement

The datasets presented in this study can be found in online repositories. The names of the repository/repositories and accession number(s) can be found in the article/supplementary material.

## Ethics statement

The animal study was reviewed and approved by animal ethics committee in Shanghai Changzheng Hospital.

## Author contributions

CW, HY, and XL designed the experiment. CW, HM, BZ, TH, HW, LW, LH, QL, WW, and YS performed the animal trials, and sample and data analysis. CW, HM, and BZ wrote the manuscript. HY and XL revised the manuscript. All authors contributed to the article and approved the submitted version.

## Funding

This study was supported by the Research Projects of Shanghai Changzheng Hospital (No.0910 and No.2020YCXYJ-ZD06) and the National Natural Science Foundation of China (No. 81572201 and No. 81802185).

## Acknowledgments

This work has benefited from GEO. We thank the GEO network for its generous sharing of large amounts of data.

## Conflict of interest

The authors declare that the research was conducted in the absence of any commercial or financial relationships that could be construed as a potential conflict of interest.

The handling editor declared a shared parent affiliation with the authors at the time of review.

## Publisher’s note

All claims expressed in this article are solely those of the authors and do not necessarily represent those of their affiliated organizations, or those of the publisher, the editors and the reviewers. Any product that may be evaluated in this article, or claim that may be made by its manufacturer, is not guaranteed or endorsed by the publisher.
